# Cryo-EM Structure and Molecular Dynamics Analysis of the Fluoroquinolone Resistant Mutant of the AcrB Transporter from *Salmonella*

**DOI:** 10.3390/microorganisms8060943

**Published:** 2020-06-23

**Authors:** Rachel M. Johnson, Chiara Fais, Mayuriben Parmar, Harish Cheruvara, Robert L. Marshall, Sophie J. Hesketh, Matthew C. Feasey, Paolo Ruggerone, Attilio V. Vargiu, Vincent L. G. Postis, Stephen P. Muench, Vassiliy N. Bavro

**Affiliations:** 1 School of Biomedical Sciences, Faculty of Biological Sciences & Astbury Centre for Structural and Molecular Biology, University of Leeds, Leeds LS2 9JT, UK; rachel.johnson@monash.edu (R.M.J.); bssjh@leeds.ac.uk (S.J.H.); m.feasey@leeds.ac.uk (M.C.F.); 2 Department of Physics, University of Cagliari, s.p. 8, Cittadella Universitaria, 09042 Monserrato, Italy; chiara.fais@gmail.com (C.F.); paolo.ruggerone@dsf.unica.it (P.R.); vargiu@dsf.unica.it (A.V.V.); 3 Biomedicine Research Group, Faculty of Health and Social Sciences, Leeds Beckett University, Leeds LS1 3HE, UK; m.patel2324@student.leedsbeckett.ac.uk (M.P.); v.l.g.postis@leeds.ac.uk (V.L.G.P.); 4 Diamond Light Source, Membrane Protein Laboratory (MPL), Diamond House, Harwell Science and Innovation Campus, Fermi Ave, Didcot OX11 0DE, UK; harish.cheruvara@diamond.ac.uk; 5 School of Life Sciences, University of Essex, Wivenhoe Park, Colchester CO4 3SQ, UK; 6 School of Biosciences, University of Birmingham, Birmingham B15 2TT, UK; r.l.marshall@bham.ac.uk

**Keywords:** *Salmonella*, multidrug efflux pump, membrane proteins, multidrug resistance, AcrB, cryo-EM, molecular dynamics

## Abstract

*Salmonella* is an important genus of Gram-negative pathogens, treatment of which has become problematic due to increases in antimicrobial resistance. This is partly attributable to the overexpression of tripartite efflux pumps, particularly the constitutively expressed AcrAB-TolC. Despite its clinical importance, the structure of the *Salmonella* AcrB transporter remained unknown to-date, with much of our structural understanding coming from the *Escherichia coli* orthologue. Here, by taking advantage of the styrene maleic acid (SMA) technology to isolate membrane proteins with closely associated lipids, we report the very first experimental structure of *Salmonella* AcrB transporter. Furthermore, this novel structure provides additional insight into mechanisms of drug efflux as it bears the mutation (G288D), originating from a clinical isolate of *Salmonella* Typhimurium presenting an increased resistance to fluoroquinolones. Experimental data are complemented by state-of-the-art molecular dynamics (MD) simulations on both the wild type and G288D variant of *Salmonella* AcrB. Together, these reveal several important differences with respect to the *E. coli* protein, providing insights into the role of the G288D mutation in increasing drug efflux and extending our understanding of the mechanisms underlying antibiotic resistance.

## 1. Introduction

Bacterial multidrug resistance (MDR) is a growing global concern with many antibiotics now ineffective against major classes of pathogens. This is especially pertinent in Gram-negative organisms, which are intrinsically more resistant due to the presence of a double membrane. *Salmonella* is an important Gram-negative pathogen, the prevalence of which is increasing especially in nosocomial settings. While there are many adaptive routes for the development of MDR in *Salmonella* [1], the role of the multidrug efflux-pumps in the process has been identified as one of the key mechanisms for escaping the antibiotic and biocide selective pressures [2,3,4]. The action of the resistance-nodulation-division (RND) transporter AcrB provides the principal antimicrobial resistance efflux function in *Salmonella enterica* serovar Typhimurium (from here on, *S.* Typhimurium) [5]. In vivo AcrB forms a complex with the outer membrane factor (OMF) TolC and the periplasmic adaptor protein (PAP) AcrA, forming a functional tripartite complex with a 3:6:3 stoichiometry respectively, that spans both the inner and outer membrane [3,6,7,8,9]. Among the nine different tripartite efflux pumps involved in MDR in *S*. Typhimurium [5], seven form tripartite complexes with TolC [10], and AcrB is the primary transporter associated with MDR. Consistent with its major role, the loss of AcrB confers a loss of virulence in *Salmonella* [11] despite the considerable overlap of specificities and partial compensation from overexpression of homologous RND-based tripartite-pumps [12]. Furthermore, the presence of the AcrAB-TolC efflux pump is required for the development of ciprofloxacin resistance [13] and is essential for biofilm formation [14].

Most of our current knowledge of AcrB transporter function is based on the *E. coli* orthologue, which shares 94.7% sequence identity to the *S.* Typhimurium pump. AcrB is organised as a homotrimer [15], the functional rotation of which has been proposed as a structural mechanism of the efflux process with each protomer undergoing sequential conformational cycling of states known as access, binding and extrusion (A, B and E respectively) [16], or, alternatively, in parallel to the ATP-synthase cycle—loose, tight, open (L, T, O) [17]. Our understanding of this mechanism has been reinforced by both structural and mutagenesis approaches including conditional cross-linking [18,19,20], as well as by computer simulations [21,22]. The sequestering of substrates/drugs by the pump can occur through a number of different pathways—known as substrate channels, that allow for capture of the cargo from the periplasmic space (channel 2 and channel 3) or from the outer leaflet of the inner membrane (channel 1 and channel 4) [23,24,25]. In the L (A) protomer, the channels 1 and 2 converge on a “proximal” drug-binding pocket (PBP), also known as the “access” pocket [19,23,26], which is separated by a “switch-loop” from the deep- or distal-binding pocket (DBP). The latter becomes occupied in the T protomer. Channel 3, which originates from an interprotomer space known as the vestibule has been shown to bypass the PBP providing direct access to the DBP and a distinct selectivity [27]. Channel 4 was discovered very recently as the putative entry path for fusidic acid and other carboxylated antibiotics, and is directly connected to the DBP within the T protomer (from here on referred as DBP_T_) [25,28].

The electrochemical potential of the membrane is utilised for transport via a proton-relay located in the transmembrane (TM) portion of the protein, which induces a conformational change that is communicated to the drug binding pockets over > 50 Å via a piston-like action involving TM helix 2 [21,29]. Recent free energy calculations suggest that protonation of D408 in the TM domain of the drug-bound protomer drives the functional rotation [21], albeit all the residues forming the proton relay are essential.

Although the main mechanism of efflux-based resistance in Enterobacteriaceae is provided by overexpression of the efflux pumps via mutations of the regulatory regions of their operons or their respective repressors (e.g., in *E. coli* expression of the genes of the principal efflux pump *acrAB/tolC* is predominantly controlled by the interplay of the local and global transcription regulators AcrR and MarA, while in *Salmonella,* these genes are under the control of AcrR and RamA, respectively, for reference see [30,31,32]), a new mechanism of adaptive resistance has been reported, namely the selection of mutations altering the specificity of the RND-transporter itself [33]. The particular mutation, which arose during the antibiotic treatment of a patient with a complex *Salmonella* infection [34,35], has resulted in a substitution, G288D, within the *Salmonella* Typhimurium AcrB transporter (hereafter *S*TmAcrB_G288D_). This mutation, is responsible for the occurrence of MDR, particularly to ciprofloxacin, increasing the MIC to the drug over 60 folds. Thus, unveiling the molecular structure of *S*TmAcrB and the role of specific residues is critical to our understanding of the mechanism of antibiotic recognition and efflux that confers the clinically relevant MDR in *Salmonella*, yet to date there has not been an experimental structure of *Salmonella* AcrB available.

Here, we report the first structure of the *Salmonella* AcrB transporter carrying the clinically relevant G288D mutation, solved by cryogenic electron microscopy (cryo-EM). This provides an insight into the structural rearrangement AcrB and also the role of the G288D mutation that underlies its multidrug resistance. Molecular dynamics (MD) simulations were further employed to provide atomistic details on the alteration of protein functional dynamics due to the G288D substitution.

## 2. Materials and Methods

### 2.1. Cloning of AcrB G288D

DNA library from clinical isolate strain L18 [33], was used to amplify *Salmonella* Typhimurium AcrB G288D mutant (*S*TmAcrB_G288D_) using the following primers:

Salm_acrB+: TAGCTTCATATGCCTAATTTCTTTATCGATCGCCCTATATTTGCGTGGGTG

Salm_acrB−: ATATTCCTCGAGGCGATGTTCTGTCGAATGACTATGCTCAATATCTTCGC

The PCR product was cloned into pET26b using the *NdeI* and *XhoI r*estriction sites (underlined). BW25113 was used as the propagating strain for cloning. *E. coli* C43ΔacrB (DE3) cells were used for protein expression.

### 2.2. Protein Overexpression and Purification

The protein was expressed using auto-induction and membrane preparation, styrene maleic acid (SMA) solubilisation and protein purification were performed using the protocols previously reported [36,37]. Cells were disrupted with a cell disrupter (Constant Systems Ltd.) at 30 kpsi after resuspension in 20 mM Tris, 0.5mM EDTA pH 7.9 (4 °C) in a ratio of 4:1 buffer to pellet. The cell membranes were isolated by differential centrifugation as previously described [38]. AcrB was extracted in its native trimeric form (~340kDa) using 2.5% (*w*/*v*) SMA, incubated for 2 h at room temperature as previously described [36,39]. The insoluble material was removed by centrifugation (100,000× *g*, 1 h, 4 °C). The supernatant was mixed with 2 mL HisPur™ Cobalt Resin (Thermo scientific) and incubated in batch overnight at 4 °C with gentle agitation. Following packing, the resin was washed twice with wash buffer (50 mM Tris-HCl, 500 mM NaCl, 10% (*w*/*v*) glycerol). After elution (50 mM Tris-HCl, 500 mM NaCl, 10% (*w*/*v*) glycerol, 300 mM imidazole), the peak fractions were pooled out and dialysed against a buffer free of imidazole.

### 2.3. Negative Stain Electron Microscopy

The purified *S*TmAcrB_G288D_ sample was assessed for sample quality by negative stain microscopy and grids were prepared as previously described [39]. Briefly, carbon-coated grids were glow-discharged for 40 s, following which 3µl of the *S*TmAcrB_G288D_ (~20 µg/mL) was added to the grid and stained with 2% uranyl acetate. Grids were imaged on a Technai F20 microscope fitted with a field emission gun operating at 200kV with a nominal magnification of 50,000× on a CMOS detector.

### 2.4. Cryo-Electron Microscopy

For single particle cryo-EM analysis, gold quantifoil grids (2.1 mesh) were glow discharged (20 s) before applying 3µL of ~1 mg/mL *S*TmAcrB_G288D_. Grids were prepared using a Vitrobot Mark IV and blotted with Ash-free Whatman filter paper (No. 50) for 6 s using a blot force of 6. Data were collected on a G2 Titan Krios fitted with a Gatan K2 direct electron detector operating in counting mode at the Astbury Biostructure facility, Leeds, UK. EPU was used to setup a 72-h data collection resulting in 3210 micrographs at 1.07 Å/pixel. Motion correction was carried out in MotionCor2 and CTF values determined using Gctf [40,41]. Initial two-dimensional (2D) references were generated in RELION from manually picked particles to generate reference free classes for autopicking the remaining data [42]. Autopicking resulted in a total of 965,863 particles which were subjected to 2D classification and after removal of those belonging to poorly resolved classes ~316,000 particles remained. After further rounds of 2D and three-dimensional (3D) classification the particle number was reduced to 105,901. The 3D refinement was conducted in RELION 2.1 using a previously published cryo-EM AcrB structure (Electron Microscopy Databank (EMD)-3887), filtered to 60 Å as a starting model. Following the 3D auto-refinement and particle polishing the resulting model had a global resolution of 4.6 Å, as determined by the 0.143 cut-off criteria. The final model had an automatically determined B-factor of –305 applied within RELION 2.1 [42]. To examine the effects of the G288D mutation on the oligomerization of *S*TmAcrB_G288D_ reconstructions were generated in both C1 and C3 symmetry with the C1 symmetry reconstruction reaching a similar resolution and showing the same overall architecture. For model fitting the *E. coli* AcrB crystal structure (Protein databank (PDB) ID: 4ZLJ) was put through “threading” in Phyre 2 [43] to produce a model of the *S*TmAcrB_WT_ structure. The G288D mutation was manually edited within Coot [44]. The high sequence similarity between *E. coli* AcrB and *S*TmAcrB_G288D_ resulted in a model of high confidence. The resulting *S*TmAcrB_G288D_ model was subsequently docked in Chimera followed by rigid body refinement using the MDFF program [45]. The model then underwent model refinement in Coot and real space refinement in PHENIX. Data collection and refinement statistics are given in Table S1. The *S*TmAcrB_G288D_ highest resolution C3 map and fitted structure have been deposited within the Electron Microscopy Database (EMDB) with accession number 4460 and 6Z12.

### 2.5. Methods for Structural Analysis

Bioinformatic analysis: Multiple sequence alignments were prepared using *MAFFT* as implemented in MAFFT v.7 server (https://mafft.cbrc.jp/) [46]. ESPript 3.0 was used for structural annotations of the alignments [47]. Model superposition and additional visualisation were performed in Coot [44] and PyMOL (PyMOL Molecular Graphics System, Version 1.71 Schrödinger, LLC).

### 2.6. Homology Modelling

Three homology models were built of *S*TmAcrB_WT_ and *S*TmAcrB_G288D_ using different X-ray crystal structures of wild type AcrB from *E. coli* (hereafter *Ec*AcrB_WT_) (PDB IDs 4DX5, 4DX7 [19] and 2J8S [48]) as templates. The amino acid sequences of both *Ec*AcrB_WT_ and *S*TmAcrB_WT_ were obtained from the Uniprot database (Uniprot IDs: P31224 and Q8ZRA7, respectively). The absence of gaps was verified through a sequence alignment with ClustalOmega [49]. The homology models were generated using Modeller 9.3 [50], each having a MOLPDF score greater than 1.5× 10^5^, and included the full range of residues (1–1033) in every template. The homology models of *S*TmAcrB_G288D_ were further energy-minimized into the experimental C1 cryo-EM map presented in this work with the program Flex-EM [51]. We performed structural optimization of the models for up to 40 iterations, and we ranked the final structures based on their cross-correlation function (hereafter CCF) (see Table S2).

### 2.7. Molecular Dynamics Simulations

The homology models of *S*TmAcrB_WT_ and *S*TmAcrB_G288D_ were used as starting structures to perform all-atom MD simulations. Following previous work [33,52], we simulated the truncated structure including only the porter domain and a few residues at the interface with the TM domain (namely, residue segments 32-335 and 564-870), imposing positional restraints on the C*α* atoms of the residues found within 5 Å from the bottom of the structure (weight of the restraints: 1 kcal/mol). Those residues involved were in Subdomain PN1 (V32, A33, Q34, T37, I38, and A39), subdomain PN2 (A297, N298, A299, T330, P331, and F332), subdomain PC1 (L564, P565, D566, K632, D633, W634, P638, G639, E640, A670, I671, V672, T676, A677, and T678) and subdomain PC2 (P710, D711, L712, G838, E839, A840, Q865, E866, and R867). The selected portion of the protein was inserted in a truncated octahedron filled with 0.15 KCl aqueous solution, setting the minimum distance between the protein and the edge of the box to 16 Å. The topology and the initial coordinate files were created through the *leap* module of AMBER18 [53]. Protein and water were represented using the ff14SB force field [54] and the TIP3P model [55], respectively, while the parameters for the ions were retrieved from [56]. The system was enclosed in a truncated octahedron filled with 0.15 M KCl aqueous solution, and the minimum distance of the protein and the border of the box was set to 16 Å. The MD simulations of each system were done according to the following procedure. Firstly, we performed a multi-step structural relaxation combining steepest descent and conjugate gradient methods, using the *pmemd* module of AMBER18 [53], as described in previous publications [52,57,58,59]. The relaxation was followed by two MD simulations runs to heat the system from 0 to 310 K: i) from 0 to 100 K in 1 ns under constant-volume conditions and with harmonic restraints (k = 1 kcal·mol^−1^·Å^−2^) on the heavy atoms of both the protein and the lipids; ii) from 100 to 310 K in 5 ns under constant pressure (set to a value of 1 atm) and with restraints on the heavy atoms of the protein and on the z coordinates of the phosphorous atoms of the lipids to allow membrane rearrangement during heating. Next, we performed a series of 10 equilibration steps to equilibrate the box dimensions. Each step was of 100 ps in duration (total 1 ns) and was carried out under isotropic pressure scaling conditions through the Berendsen barostat. The Langevin thermostat was also used to maintain the temperature constant, with a collision frequency of 1 ps^−1^. Finally, for every system we performed three independent MD simulations, each with a production run of 150 ns in length. Time steps of 0.5 fs and 2 fs were used during the heating and equilibration stages, respectively. In the production run a time step of 4 fs was adopted under an isothermal-isobaric ensemble after hydrogen mass repartitioning [60]. Moreover, the lengths of all the R-H bonds were constrained with the SHAKE algorithm. Coordinates were saved every 100 ps. Long-range electrostatic forces were evaluated with the particle mesh Ewald (PME) algorithm, with a non-bonded cut-off of 9 Å.

### 2.8. Post-Processing of MD Trajectories

The MD trajectories of *S*TmAcrB_WT_ and *S*TmAcrB_G288D_ were firstly processed by performing a cluster analysis with the *cpptraj* module of AMBER18 [53]. For each trajectory, we considered only the last 140 ns of the production run, where the RMSD of the protein with respect to the first frame is fairly constant (Figure S1). Every trajectory was subjected to three clustering procedures, in each of which the distance-RMSD metric was applied to the DBP of a different monomer of AcrB, generating 100 clusters. In this way, we obtained 300 clusters per trajectory, divided in three equal subsets (1 subset per monomer). For each subset, the representative centroid structures of all clusters were used to perform several analyses aimed at assessing how the size and shape of the DBP are affected by the G288D mutation. To this end, we firstly estimated its volume of in the L, T, and O monomers of both *S*TmAcrB_WT_ and *S*TmAcrB_G288D_.

The same analysis was then performed on 5 experimentally derived crystal structures of the *Ec*AcrB_WT_, which were chosen as reference structures to identify variations between *E. coli* and *S*TmAcrB_WT_. These structures have PDB IDs 4DX5, 4DX7 [19], 2J8S [48], 2I6W (the last being a symmetric LLL structure) [61] and 6BAJ (the structural model recently derived from cryo-EM data by Qiu et al.) [62]. The volume calculations were performed using the POVME 2.0 software [63], adopting a grid spacing of 0.5 Å. Additional analyses were conducted to better characterize of the impact of the G288D mutation. These included the calculation of the gyration radius of the DBP, the number of (pseudo)contacts between the PC1 and PC2 subdomains and the number of waters in the first and second solvation shell of residue 288. Such analyses were conducted on every protomer of AcrB. Calculations of the radius of gyration and of the number of (pseudo)contacts were carried out using in-house *tcl* scripts and performed on the cluster representatives of *S*TmAcrB_WT_ and *S*TmAcrB_G288D_, as well as on the reference structures of the *Ec*AcrB_WT_. The radius of gyration was computed for three different regions of the DBP: the whole DBP (S46, Q89, S128, E130, E134, F136, V139, Q176, L177, F178, S180, E273, N274, D276, Y327, L573, F610, V612, R620, F628), the hydrophobic trap (hereafter HP trap) (F136, V139, F178, Y327, L573, F610, V612, and F628) and the upper DBP (S46, Q89, S128, E130, Q176, L177, G179, S180, E273, N274, D276 and R620). As to the number of (pseudo)contacts, it was calculated by using a distance cut-off of 10 Å among the Cα carbons of selected regions of PC1 (segment 571-667) and PC2 (segments 679-721, 822-859). Regarding the first and second water shells of residue 288, these regions were defined using distance cut-offs of 3.4 Å and 5 Å, respectively. Calculations were performed on the last 140 ns of every MD trajectory of *S*TmAcrB_WT_ and *S*TmAcrB_G288D_, using the *cpptraj* module of AMBER18 [53].

Moreover, we monitored the Loose/Tight/Open (LTO) asymmetry of *S*TmAcrB_G288D_ along the MD trajectories. To perform this analysis, we used as a reference the *Ec*AcrB_WT_ crystal structure with PDB ID 4DX7 [19], in which the protein is found in the LTO state. Thus, for each frame in the last 140ns of the MD production run, we calculated the RMSD of each conformer of the mutant with respect to every conformer of the *E. coli* reference structure. Only the C_α_ atoms were considered for this calculation.

## 3. Results

### 3.1. Oligomeric State and Overall Fold of S. Typhimurium AcrB

**Figure 1:**
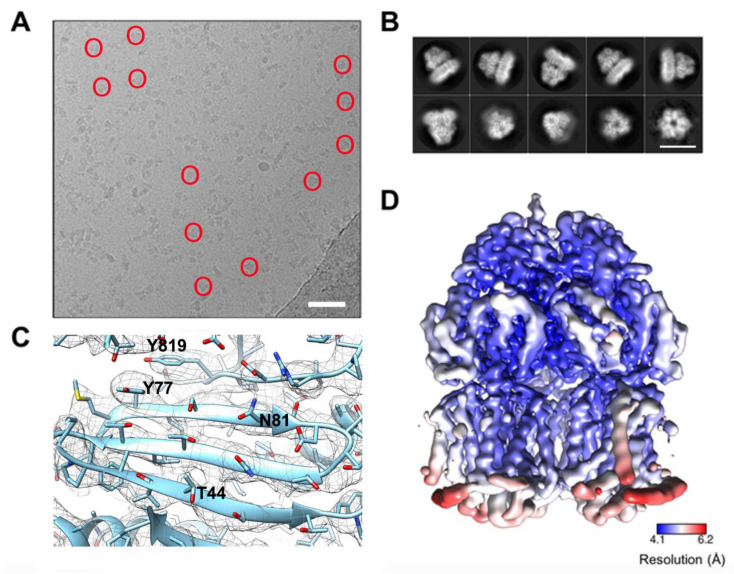
Single particle cryo-electron microscopy reconstruction of STmAcrB G288D processed in C3 space group. (**A**) Representative micrograph of *S*TmAcrB G288D collected on a K2 camera, with a scale bar in white representing 50 nm. Some representative STmAcrB G288D particles are circled in red. (**B**) Two-dimensional (2D) classes of *S*TmAcrB G288D, produced in RELION as seen from the side, high angle, and top views. The scale bar represents 15 nm. (**C**) Representative experimental electron map density processed in C3 space group (mesh), with the refined *S*TmAcrB G288D model fitted (cyan). Reference residues labelled within the map include, T44, Y77, N81 and Y819. (**D**) The C3 derived map from *S*TmAcrB G288D coloured by local resolution in Angstroms (Å) showing the core to be more highly resolved, while the transmembrane flanking regions display lower resolution.

Here we report the first ever experimentally-derived structure of the AcrB from *S.* Typhimurium. *S*TmAcrB_G288D_ structure was determined by single particle cryo-EM to a global resolution of 4.6 Å. *S*TmAcrB_G288D_ was expressed in an *E. coli* strain deficient of the native AcrB transporter (C43ΔacrB(DE3)) to ensure only the *Salmonella* orthologue was expressed. For protein extraction a styrene maleic acid (SMA) co-polymer approach was used, which we have previously shown to be effective at isolating the *E. coli* AcrB in sufficient levels for both negative-stain and cryo-EM analysis [36,37,39]. Negative stain and the subsequent cryo-EM sample (Figure 1A) showed a mono-disperse sample with no significant aggregation or degradation.

In contrast to our previous studies of *Ec*AcrB by single particle cryo-EM here we have collected significantly more data to improve the resulting resolution and quality of the map [36]. The micrographs showed a monodisperse sample with variation in the orientation within the ice (Figure 1A). The resultant classes revealed clear secondary structure detail for both side and top views (Figure 1B). As expected, based on the high sequence similarity, the overall architecture when processed in C3 symmetry is similar to that of *E. coli* AcrB and the functional biological unit of the protein is formed by three protomers displaying overall 3-fold symmetry [15] (Figure 1B). The transmembrane domain of each protomer is formed by 12 α-helices with a single α-helix flanking the base of the transmembrane domain. All the α-helices were well-defined in the cryo-EM map and could be placed with high confidence. Although additional density can be seen between the helices the resolution is not sufficient to assign any lipids or SMA-polymer with confidence. The periplasmic domain is well-resolved and shows features consistent with a local resolution of ~4.1 Å. Both α-helices and β-strands are well-resolved and bulky amino acid side-chains can be fitted within the map with confidence (Figure 1C). Analysis of the local resolution in C3 map shows the core to be more highly resolved with lower resolution features displayed on the exterior of the complex, especially within the transmembrane region (Figure 1D).

### 3.2. Comparison between Salmonella AcrB G288D Processed in the C3 Space Group and the WT E. coli Transporters

The overall structure of *S*TmAcrB is closely related to that of the previously reported *E. coli* orthologue [15], which is consistent with the high level of sequence conservation between the two transporters. Sequence analysis reveals a 94.7% identity over the 1048 residues as calculated by the SIM Alignment tool (https://web.expasy.org/sim/) [64], (Figure S2).

**Figure 2:**
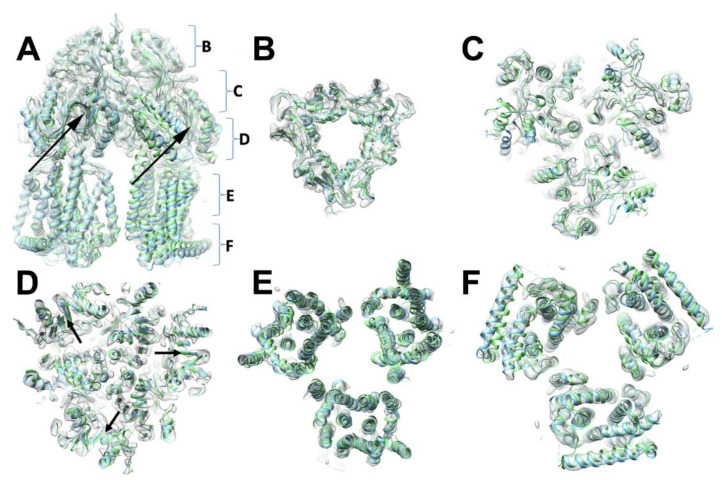
Comparison of *E. coli* and *S.* Typhimurium AcrB three-dimensional (3D) structures. (**A**). A side-view of the trimer of the *S*TmAcrB G288D refined model (cyan) superposed over the *E. coli* orthologue (*Ec*AcrB) (light green) and fitted into the experimental electron density map (semi-transparent grey). (**B**–**F**) Horizontal slices through the *S*TmAcrB C3 cryo-EM electron-density map from the top (**B**) to base (**F**). Both *S*TmAcrB G288D and *Ec*AcrB are fitted within the density. The relative positions of the slices are indicated in (**A**). The position of G288D mutation within the structure is indicated with black arrows in (**A**) and (**D**).

**Figure 3:**
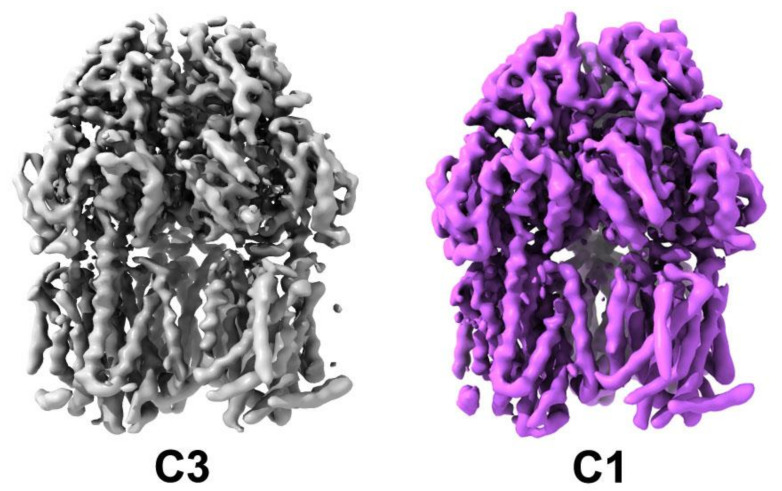
Comparison of *S*TmAcrB G288D cryo-EM maps refined in C3 and C1 with no difference in the overall architecture observed but slightly improved resolution in the C3 map.

The position of the secondary structure elements, and correspondingly the (sub)domain organisation is preserved, as is to be expected given there are no significant additional residue inserts between the sequences (Figure 2; Figure S2). Consistently, the refined trimer preserves its central symmetry, but there are slight discrepancies between the individual protomers which amount to an RMSD of 0.35–0.38 Å over the C-alpha backbone. This small change results from each chain being fitted and refined within the map without the enforcement of threefold symmetry, reflective of the modest (~4.6 Å) resolution within the map. While symmetric structures in the R32 space group have been produced both in apo- (1IWG.pdb, [15]), and in a linezolid-bound form of *Ec*AcrB (4K7Q.pdb, [65]), as mentioned in the *Introduction*, in its functional state AcrB presents a breakdown of the central-axis three-fold symmetry (with conformers referred to as either L, T, O (Loose, Tight, Open) [17]; or A, B, E (Access; Bound; or Extrusion) [16]), which is also observed in the associated X-ray structures. The related *Ec*AcrB cryo-EM structure (6CSX.pdb, [62]) also displayed a clear breakdown in the 3-fold symmetry related to the presence of defined states within the complex. Therefore, we also refined *S*TmAcrB_G288D_ in the C1 space group corresponding to an asymmetric trimer resulting in a map which closely resembled that of the C3 but with small local changes, consistent with the change in state of each protomer (Figure 3).

To establish whether the functional rotation states are also preserved in the *S*TmAcrB_G288D_ we set out to analyse which of the reported *Ec*AcrB protomer-states our structure resembled most closely. Pairwise superpositions using the high-resolution asymmetric structure 4DX7 [19], resulted in a RMSD over the *S*TmAcrB backbone in the ranges of 1.69–1.75 to 1.54–1.64 and to 1.51–1.54 Å (against the L, T, and O protomers, respectively), while the superposition with the symmetric 4K7Q yielded a lower RMSD of 1.03–1.08 Å. Note that superposition of 4K7Q onto 4DX7 chains yielded RMSDs corresponding to 1.19, 1.26 and 1.40 Å (for the L, T, and O conformers, respectively). The above indicates that the while asymmetric, the conformation observed within our cryo-EM *S*TmAcrB structure deviates only moderately from the symmetrised *E. coli* protomer state.

### 3.3. Implications for Drug Selectivity and Efflux Efficiency

**Figure 4:**
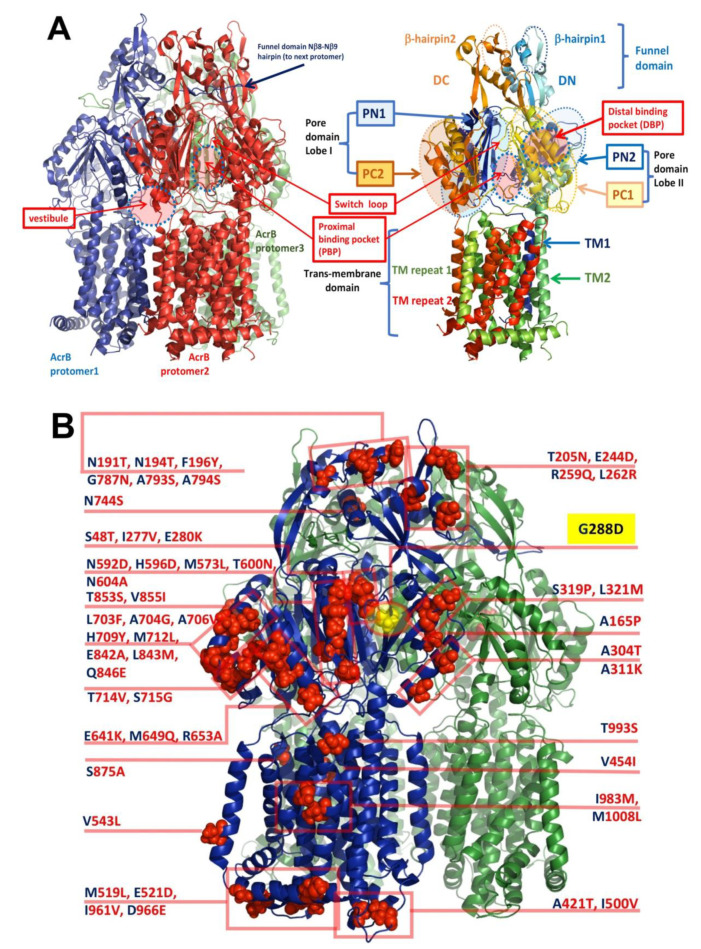
(**A**) General organisation of the *S*TmAcrB G288D trimer and detailed sub-domain organisation of the single protomer. (**B**) Mapping of the sequence differences of *S*TmAcrB_G288D_ vs. *Ec*AcrB_WT_ shown on one of the three protomers as red spheres. The labels indicate single-letter residue code for the differing residues in *E. coli* AcrB (in blue), alongside with the equivalent positions and substituted side-chains in *Salmonella* AcrB (in red). The location of the mutation G288D is indicated with a yellow box.

We next sought to understand how sequence differences between the *E. coli* and *S.* Typhimurium AcrB could translate into differential selectivity to antibiotics. Compared to the *Ec*AcrB, the novel *S*TmAcrB structure shows changes in the substrate pathway and the DBP, which is sandwiched between the PC1 and PN2 subdomains (Figure 4A) of the periplasmic “pore”- or porter-domain. The principal access for soluble drugs from the periplasmic space is via access-tunnel 2, which is located between the two principal lobes of the pore-domain, and is connected to the “access” or proximal binding pocket (PBP), which also receives the membrane-connected access-tunnel 1 [23]. As discussed in the Introduction, the DBP is separated from the PBP by the so-called “Glycine” or “switch”-loop, the flexibility of which affects the drug-passage through the latter [19,66]. As will be discussed in detail further below, the new *S*TmAcrB structure refined in C1 space group, displays asymmetry of protomers, which allows them to be assigned to the specific conformational states (L, T, O conformers) described above, however the range of structural variation between them appears to be significantly lower than the corresponding conformers within *Ec*AcrB; therefore, we analysed the sidechains based on the C3 map, which had a higher local resolution. Due to their high overall homology and lack of gaps in the alignment, for ease of presentation, below, when referring to the amino-acid differences between the *E. coli* and *Salmonella* AcrB, we refer to the divergent residues occupying the same position as “substitutions” (see Figure 4B).

Yet, despite the low overall percentage of sequence difference, the amino-acid differences cluster into several discrete groups which may have significant impact on the functional profile of the pump and its dynamics. These residue differences can be split into two broad classes based on their potential effects—one affecting the conformational dynamics of the protomer, while the second impacting the substrate recognition and processing (Figure 4B).

**Figure 5:**
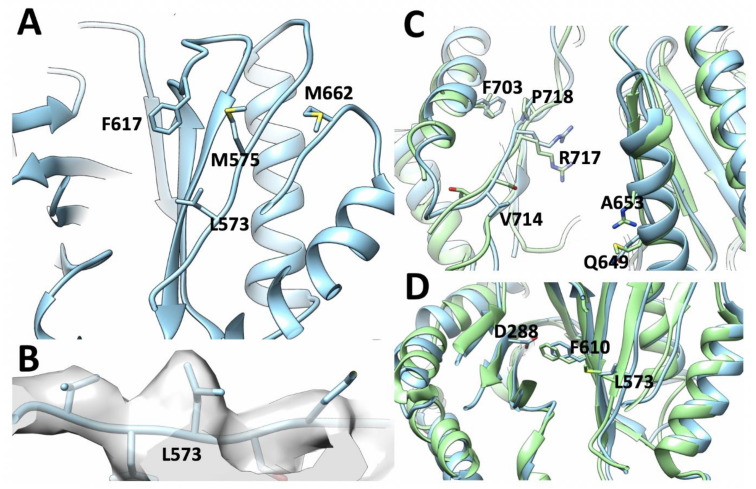
Close look of the drug-binding pockets of *S*TmAcrB G288D. (**A**) The PBP (access binding pocket) and the switch loop in *S*TmAcrB G288D with those residues that make up the larger hydrophobic “Met-cluster” shown. (**B**) Representative map density for *S*TmAcrB G288D showing the proximity of L573, which in *Ec*AcrB has a bulkier methionine allowing unambiguous placement of this sidechain. (**C**) The outer rim of the cleft between the two lobes of the porter domain which provide entry to tunnel 2. (**D**) Comparison of the structural differences in the DBP in proximity of the G288D mutation. In all panels *S*TmAcrB is shown in cyan and *Ec*AcrB in green.

Of the latter, of particular interest is the M573L substitution in the primary substrate pathway. In *Ec*AcrB M573 occupies a critical position between the proximal binding pocket and the switch loop, where it is part of a larger Met-cluster that includes M575 and M662; and, in conjunction with the F617 which belongs to the switch-loop, also provides a part of the hydrophobic barrier. The residue is well resolved in the electron-density map and the Leucine-substitution appears to cause local rearrangement of the hydrophobic residues in the so-called hydrophobic trap (HT) of the DBP, including a displacement of the ring of F617 (Figure 5A,B).

A double substitution T714V/S715G located on the outer left rim of the cleft between the two lobes of the porter domain, forming the entry of tunnel 2 leading to the proximal drug-binding pocket might plausibly impact the selectivity of some drugs. On the opposite rim of the tunnel 2 cleft there are two more neighbouring substitutions, namely M649Q plus R653A. While these latter substitutions are on the outer rim of the entrance leading to the PBP their nature suggests that they would result in a significant rearrangement of the electrostatic properties as well as water-coordination at the entrance of the pocket and thus may affect drug access (Figure 5C).

Furthermore, the double substitution L703F/A704G is able to provide a novel phenyl-ring stacking interaction with the P718, which could affect the local dynamics of the PBP entry, as these residues are located just above V714/G715 and immediately next to R717 which has been shown to participate in substrate coordination in the proximal pocket [19,23]. While the quality of the map at this region does not permit the unambiguous positioning of F703 its increased bulk must be accommodated within the structure (Figure 5C).

### 3.4. Structural Changes Attributable to G288D Mutation

An important aspect of our cryo-EM *S*TmAcrB structure refined in a C3 is the presence of a G288D mutation which confers greater resistance to fluoroquinolones [33]. However, analysis of the novel cryo-EM structure reveals that the G288D mutation brings D288 in close contact with the well-resolved sidechain of F610. Unfortunately, since negatively charged side-chains are more susceptible to radiation damage their resulting density is often poor within the map [67]. Hence, while there is no-clear density for D288 side chain, there are also no preferred rotamers that could avoid this close contact, suggesting that D288 is either in a less-favoured conformation or maybe in multiple conformations between protomers, explaining the poor density for this region. It is interesting to note that the β-strand on which G288 is located does not show a significant shift to accommodate the extra bulk of aspartate sidechain in the mutated *Salmonella* structure compared to the *Ec*AcrB_WT_ DBP (Figure 5D). However, the residues around D288 show a notable shift, which may accommodate the increased bulk and the major change in the electrostatics of the pocket associated with the increased hydration detected in our previous work [33]. In particular, F610 is positioned further away from position 288 causing an increase in pocket size (Figure 5D). Through these changes the lower part of hydrophobic pocket is locally enlarged. This expansion occurs around Q176 and F178, which have both previously been shown to participate in the coordination of substrates within AcrB [19,26,33,68].

### 3.5. Mechanism of G288D Mutation Inferred from MD Simulations

Notably, the above observations were based on the static and medium-resolution cryo-EM structure; therefore, we sought to further validate and extend our predictions by performing MD simulations starting from several independent homology models. Many different all-atom structural models of *S*TmAcrB_G288D_ and *S*TmAcrB_WT_ were derived through homology modelling, and the models of the former were minimized against the C1 cryo-EM map presented in this work. Model accuracy was evaluated through the CCF (see Materials and Methods), which, for each model, slightly improved starting from every template (Table S2). Moreover, the optimization against the cryo-EM map did not cause significant structural changes from the initial models (last column in Table S2).

Starting from these structural models, we performed three independent all-atom MD simulations for each system in order to compare structural and dynamical features of the wild type and substituted *S*TmAcrB. As described in Materials and Methods, we used a truncated model of *S*TmAcrB (largely validated in previous studies; see [69] for a recent review) containing the periplasmic portion of the protein only, as it is primarily responsible for drug binding. In fact, despite this approximation the asymmetric LTO structure remained well-preserved throughout the MD simulations, consistent with the results described previously [33,52]. In particular, cross-RMSD calculations between each conformer of our models against each conformer of the reference structure (PDB ID 4DX7 [19], see Materials and Methods) revealed that the asymmetric LTO structure is similarly retained in *S*TmAcrB_G288D_ (Table 1, below).

We first focused our analysis on the impact of the G288D substitution on the volume of the DPB (V_DBP_) across all AcrB conformers. The largest differences are seen in the volume of the distal binding pocket of monomer T (hereafter DBP_T_), which undergoes a significant expansion with respect to the WT *Salmonella* protein (Table 2). Note that while relative to the *E. coli* orthologue the V_DBP_ of the *S*TmAcrB WT is about 800 Å^3^ smaller, the effect of G288 mutant on the DBP results in an expansion of approximately 450 Å^3^ in the T-conformer bringing it closer to that of the *E. coli* orthologue.

The trend seen for V_DBP_ is confirmed by the calculation of the gyration radius (Table 3), which increased significantly only for the DBP_T_, and in particular in the HP trap, for which the increment amounts to almost 1 Å with respect to *Ec*AcrB_WT_ (of less than 0.5 Å in the other monomers).

**Figure 6:**
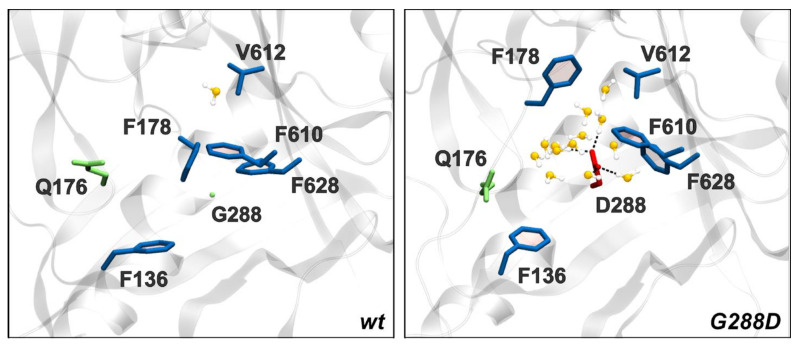
Close-up of the residue 288 and surrounding residues of the HP trap in *S*Tm*AcrB_WT_* and *S*Tm*AcrB_G288D_*. Waters belonging to the first and second hydration shell of residue 288 (distance threshold: 5 Å, see Materials and Methods) are also shown, and hydrogen bonds involving residue 288 are represented as dashed lines. This image has been created using two representative frames of MD trajectories.

In addition, the analyses performed on multiple trajectories of both the WT and substituted *S*TmAcrB transporter reveal that the number of waters in the 1st and 2nd solvation shells of residue 288 drastically increases upon mutation (Table 4 and Figure 6, below). The increased hydration of the DBP_T_ “breaks” the hydrophobic character of the hydrophobic trap (HP trap), and is likely responsible for the increased volume and gyration radius of the DBP_T_ and for the altered specificity of the transporter described previously [33]. Our findings are reasonable, as the mutation of a glycine into a charged and bulkier residue is expected to have the largest impact on the structure, dynamics, hydration of the surrounding (prevalently hydrophobic) region. Moreover, they agree with previously published results [33], although here we have extended the analyses to conformers other than T and we have increased confidence by using multiple and independent structural models of AcrB.

Along these lines, in addition to the impact of the mutation on DBP_T_, we also evaluated here the occurrence of structural changes in the other main binding pocket of AcrB, the PBP of the L conformer (hereafter PBP_L_). This site is found beneath the entrance cleft [24] and involves subdomains PC1 and PC2. The calculation of the volume of the PBP is less-straightforward due to its relatively open architecture and somewhat vague definition of boundaries. Therefore, to quantify the changes occurring in the region we evaluated the number of contacts between domains PC1 and PC2, which should reflect the opening/closing of the external cleft leading to the access pocket. Indeed, within representative *E. coli* structures, such as 4DX7, the number of average contacts increases from monomer L to T and to O. Rather surprisingly we found that these subdomains lose contact in the G288D *S*TmAcrB variant compared to the WT protein (Table 5, below), and this especially affects the L-conformer. This results in L-conformer being more open towards the periplasm and thus providing easier entrance for effluxed compounds.

## 4. Discussion

Despite its clinical importance, the structure of *Salmonella* AcrB has remained poorly understood until now. This could partially be due to the fact that while the *E. coli* AcrB readily crystallises and has been described as a persistent contaminant of both 2D and 3D crystallization screens even at picogram amounts [70,71], the *Salmonella* transporter proved rather challenging for crystallization via traditional approaches, which is unexpected considering the close homology between their sequences. This may partially be explained as *Ec*AcrB crystallises in both the H32 or C121 space groups and in both these instances the regions which form the crystal contacts differ significantly between *Ec*AcrB and *S*TmAcrB.

To circumvent these issues, here we set-out to employ a cryo-electron microscopy approach to the solution of structure of *S*TmAcrB G288D. Furthermore, to minimise structural artefacts we decided to pursue the determination of *S*TmAcrB in a native-like membrane environment, by utilizing styrene maleic acid (SMA) copolymer [37] for membrane extraction and direct solubilisation of the protein from bacterial membranes. The SMA has many benefits over traditional detergents, the main one being that it allows for a one-step extraction from cell membrane allowing the retention of native structural lipids. We have shown previously the applicability of the SMA-approach, especially in relation to AcrB for both negative stain [39] and cryo-EM structure determination [36], the latter work reaching sub-9 Å resolution. Optimisation of sample preparation allowed us to push the resolution to near 4 Å (in the periplasmic porter domains) of *S*TmAcrB, allowing confident placement of most side-chains. Our new *S*TmAcrB structure adds to only a few available recent native-lipid membrane structures of RND-transporters including the *Acinetobacter baumannii* AdeB [72] and *Ec*AcrB [62].

The transmembrane domain shows a high degree of similarity between the two models with no significant changes in helices position that could not be attributed to differences one would expect from a lower resolution model. The base of the transmembrane domain shows subtle movement of the helices at the base of the membrane (Figure 2E). This helix is clearly resolved within the EM map and can be placed with confidence although it is more poorly resolved than the core helices, consistent with some degree of mobility of this element, likely due to its position on the periphery of the complex.

### On the DBP Pocket and the Effect of G288D

The resolution of the structures obtained by cryo-EM often does not allow to investigate subtle but possibly relevant conformational changes between the WT and G288D variant of *S*TmAcrB, nor does it capture the dynamic properties of the respective molecules (and of their dynamic interaction with the solvent and the membrane) that ultimately translate into their functional characteristic.

These dynamic changes, which could be of particular interest at putative binding sites and entrance/exit gates for substrates and/or inhibitors, were thus investigated by means of full-atomic molecular dynamics simulations. The impact of the G288D substitution was expected to be most pronounced for the DBP, where it was predicted to localise. Indeed, several previous high-resolution structures of *Ec*AcrB [19,48] demonstrate that G288 sits in the middle of a β-strand beneath F178, a residue which is critical for coordination of hydrophobic drugs in the DBP. Moreover, the side chains of neighbouring residues point away from the G288D substitution (as seen in Figure 5 and Figure 6), suggesting that the side chain of the substituted aspartate residue will protrude into the pocket cavity, thus having a major effect on the coordination of potential substrates [33].

The role of mutations within the drug-binding pockets of RND transporters have only recently been appreciated as a possible additional mechanism of drug resistance [33,73,74], although earlier reports exist [75]. The G288D substitution has been demonstrated to alter the antibiotic resistance profiles for clinical isolate of *Salmonella*, notably increasing resistance to fluoroquinolones. The new cryo-EM structure, alongside the computational data presented here sheds further light on the possible mechanism of action of G288D substitution. Building on the previous work on the G288D [33], here we expanded the scope and the statistical significance of the analysis and moreover, we describe the impact of the mutation on all protomers of the transporter by means of additional analyses not performed in previous publications. Interestingly, a significant compression of the DBP_T_ was seen in *S*TmAcrB_WT_ as compared to the value calculated from the X-ray structures of *Ec*AcrB orthologue in the MD simulations. The analysis of our MD simulations suggests that the DBP of *S*TmAcrB is significantly less-voluminous than the *E. coli* orthologue, pointing to an increased steric hindrance within the pocket, which might translate into altered substrate-processing kinetics and specificities between the two transporters. However, it should be noted that such a compression was also seen in previous MD simulations of *Ec*AcrB in the absence of any ligand within the pocket, pointing to the possibility of unresolved ligands within the DBP_T_ of these experimental structures [58] although further analysis of this feature is beyond the scope the current study. Despite that, the MD analysis suggests that DBP of WT *S*TmAcrB exhibits markedly different dynamic properties to either the *S*TmAcrB D288G or *Ec*AcrB, both of which show similar, larger volumes in the T-conformer (Table 2 and Table 3). This may translate into altered specificity and increased efflux of the substrates that bind in the DBP. Indeed, changes in the DBP characteristics have been reported to account for the discrepancies in the substrate specificity of other RND-transporters e.g., AcrB and AcrD; MexB and MexY [26,58,76]. Such effect may be further enhanced by the observed loss of contacts between PC1 and PC2 of the L-conformer (Table 5), resulting in a conformation of the PBP that is more-open to the periplasm and hence more accessible for potential substrates. Thus, the significant DBP and PBP rearrangements observed in the structure and in the MD simulations likely account for the discrepancies between the WT and G288D variants, and its increased pumping efficiency for ciprofloxacin. It is worth noting however, that concurrently to increased fluoroquinolone resistance the G288D causes an increased susceptibility to doxorubicin and minocycline [33].

While there are still open questions as to the role played by the G288 within the functional cycle of AcrB, and so far the G288D mutation is the only mutation affecting this codon resulting in multidrug resistance [33], it is notable that mutations at this position have been reported more than once, both from *Salmonella* and *E. coli* [33,68,77]. The study by Soparkar et al., [68] was searching for spontaneous gain-of-function revertants compensating for the critical substitution F610A [78] affecting the packing of the hydrophobic trap within the DBP, while the Schuster et al., [77] isolated a G288S mutation in response to exposure of cells to the efflux pump inhibitor (EPI) 1-(1-Naphthylmethyl)-piperazine (NMP), strongly suggesting the residue has an important structural role affecting the DBP. Our present data further reinforces the importance of G288, highlighting not only changes of the DBP volume and conformation, but also subtle yet tangible conformational rearrangements which radiate from the mutation point (Figure 6, Table 4) affecting the packing of subdomains PC1 and PC2 and by proxy the proximal binding pocket (PBP). As reported above, these might be communicated via different arrangements of the switch-loop [24] and of the loop connecting PC2 to the funnel domain, which move closer in *S*TmAcrB_G288D_ compared to *S*TmAcrB_WT_. Thus, the G288D mutation appears to impact the plasticity of the drug-binding pocket and drug-transport pathway. These results are further corroborated by the recent analysis of *S*TmAcrB_G288D_ sensitivity to another EPI, namely phenylalanine-arginine-β-naphthylamide (PAβN) [79], which not only demonstrates that PAβN effectively inhibits both WT and G288D version of the *Salmonella* AcrB, but that that ciprofloxacin and PAβN can stably occupy the DBP at the same time. This implies that PAβN potentiates antibiotic activity by restraining drug-binding pocket dynamics, rather than preventing antibiotic binding. Hence, further analysis and docking utilising the new structure reported here would be invaluable to further elucidate the exact effects of the mutation on the efflux, and inform the design of new EPIs.

In addition to elucidating the direct effect of G288D mutation, the cryo-EM structure of *S*TmAcrB also highlights several important differences in the residues lining the principal drug-entrance tunnel 2 access leading to the PBP affecting its electrostatics (Figure 4B), as well as identifies a possible role for the M573, a residue in the control of coordination of residues at the upper-side of the DBP. Taken together these modifications and rearrangements likely account for the subtle differences between the specificities and efflux properties of *Ec*AcrB and *S*TmAcrB.

## 5. Conclusions

AcrB is a key transporter contributing to multidrug resistance and recent studies have provided significant developments in our understanding of its function. Through the study of different species and sequence variants we can start to better understand how these mechanisms are conserved across the wider RND-transporter family.

Increases in MDR in *Salmonella* Typhimurium are transforming this microorganism into a growing health threat globally, which is progressively difficult to treat. Here we have used a single particle cryo-EM approach to solve the structure of the *S*TmAcrB G288D mutant derived from a clinical isolate strain. Consistent with the high sequence similarity between the *S*TmAcrB and its well-characterised *E. coli* orthologue, the structural similarity between the two proteins is high, without significant changes in overall architecture. However, there are subtle differences present within the structure that could account for the changes in substrate transport. Indeed, computational modelling followed by MD-simulations highlighted specific features of the two transporters at their respective putative substrate-interaction sites. We also further clarify the effect of G288D mutation on *S*TmAcrB, and report that it results in a subtle expansion of the DBP pocket and may thus affect the dynamics of the pocket alongside the interprotomer-interactions within the *Salmonella* AcrB trimer. While the significance of the specific substitutions within the DBP of the *S*TmAcrB might require further investigation, the availability of the experimental structure of this clinically important protein provides the platform to inform future structural analysis and structure-driven design of potential novel efflux-pump inhibitors.

## Figures and Tables

**Table 1:** Cross-RMSD of each chain of *S*TmAcrB_G288D_, with respect to every conformer of the *Ec*AcrB crystal structure 4DX7. For each model, the calculation was performed on the last 140 ns of the production run (see Materials and Methods); the reported values correspond to the average RMSD and its standard deviation in the Loose (L), Tight (T) and Open (O) conformer. Only the C_α_ atoms were considered for this calculation.

Homology Model	Chain	RMSD (Reference Structure: *EcAcrB* 4DX7)		
		L-Conformer	T-Conformer	O-Conformer
1 (template: 2J8S)	A	2.1 (0.1)	2.7 (0.1)	3.2 (0.1)
	B	3.0 (0.1)	2.3 (0.1)	4.1 (0.1)
	C	3.5 (0.1)	3.9 (0.1)	2.2 (0.1)
2 (template: 4DX5)	A	2.4 (0.1)	3.1 (0.1)	3.0 (0.1)
	B	3.0 (0.1)	2.2 (0.1)	3.9 (0.1)
	C	3.2 (0.1)	3.9 (0.1)	2.2 (0.1)
3 (template: 4DX7)	A	2.1 (0.1)	2.8 (0.1)	3.4 (0.1)
	B	3.1 (0.1)	2.3 (0.1)	4.1 (0.1)
	C	3.1 (0.1)	3.5 (0.1)	2.1 (0.1)

**Table 2:** Values of the volume of the deep- or distal-binding pocket (DBP) (standard deviations in parentheses) in each conformer of AcrB, measured on the *E. coli* AcrB_WT_ reference structures and on the molecular dynamics (MD) trajectories of *S.* Typhimurium AcrB_WT_ and AcrB_G288D_ (see Materials and Methods).

System	Volume of DBP [Å^3^]		
	L-Conformer	T-Conformer	O-Conformer
*Ec*AcrB_WT_^1^	763 (86)	2315 (76)	1094 (76)
*S*TmAcrB_WT_^2^	957 (93)	1534 (163)	991 (96)
*S*TmAcrB_G288D_^2^	807 (78)	1979 (72)	1151 (64)

^1^ calculated on experimental reference structures; ^2^ calculated on representatives of each of the 100 clusters extracted from the MD trajectories.

**Table 3:** Radius of gyration of the DBP calculated for every AcrB conformer. The three regions of the DBP considered in this calculation are indicated as Whole (entire DBP), Upper (upper part of the binding site), and HP trap (hydrophobic trap) (see *Materials and Methods* for the definition of these regions).

System	Radius of Gyration [Å]								
	L-Conformer			T-Conformer			O-Conformer		
	Whole	Upper	HP Trap	Whole	Upper	HP Trap	Whole	Upper	HP Trap
*Ec*AcrB_WT_^1^	10.2 (0.1)	9.0 (0.1)	6.4 (0.3)	10.8 (0.1)	9.4 (0.1)	7.1 (0.3)	10.6 (0.1)	9.9 (0.1)	6.2 (0.0)
*S*TmAcrB_WT_^2^	10.5 (0.1)	9.1 (0.1)	6.3 (0.2)	10.7 (0.2)	8.7 (0.1)	6.9 (0.2)	10.9 (0.1)	9.8 (0.1)	6.3 (0.2)
*S*TmAcrB_G288D_^2^	10.5 (0.1)	9.2 (0.1)	6.4 (0.1)	11.2 (0.1)	9.2 (0.2)	8.0 (0.2)	11.0 (0.1)	9.9 (0.1)	6.5 (0.2)

^1^ calculated on experimental reference structures. ^2^ calculated on representatives of each of the 100 clusters extracted from the MD trajectories.

**Table 4:** Number of waters in the first and second solvation shell around residue 288 (in DBP), in *S*TmAcrB_WT_ and *S*TmAcrB_G288D_. The two solvation shells were defined by using a distance cut-off of 3.4 Å and 5.0 Å, respectively.

System	AcrB Conformer		
	L-Conformer	T-Conformer	O-Conformer
# 1st solv. shell waters			
*S*TmAcrB_WT_	-	0.1 (0.3)	0.0 (0.1)
*S*TmAcrB_G288D_	-	6.3 (0.6)	3.1 (0.3)
# 2nd solv. shell waters			
*S*TmAcrB_WT_	0.0 (0.1)	0.5 (0.4)	0.2 (0.2)
*S*TmAcrB_G288D_	0.2 (0.3)	11.3 (1.2)	5.7 (0.5)

**Table 5:** Number of contacts between the subdomains PC1 and PC2 in the three conformers of AcrB. Two residues have been considered in contact if the distance between their C_α_s is below 10 Å (see Materials and Methods).

System	Number of Contacts (PC1-PC2)		
	L-Conformer	T-Conformer	O-Conformer
*Ec*AcrB_WT_^1^	9 (5)	10 (2)	38 (2)
*S*TmAcrB_WT_^2^	12 (4)	6 (2)	38 (7)
*S*TmAcrB_G288D_^2^	1(1)	7(2)	31 (7)

^1^ calculated on experimental reference structures. ^2^ calculated on representatives of each of the 100 clusters extracted from the MD trajectories.

## Supplementary Materials

Supplementary materials can be found at https://www.mdpi.com/2076-2607/8/6/943/s1, Figure S1. Protein RMSD calculated along the MD trajectories of *S*TmAcrB_G288D_ aligned to the *S*TmAcrB_WT_; Figure S2. Multiple sequence alignment of *S*TmAcrB_G288D_ aligned to the *S*TmAcrB_WT_ and *Ec*AcrB_WT_; Table S1. Data collection, processing and model fitting statistics.; Table S2 Values of the Cross-Correlation Function obtained through Flex-EM for the homology models of *S*TmAcrB_G288D_, before and after the optimization inside the cryo-EM map.
