# Supplementary material for: Cryo-EM Structure and Molecular Dynamics Analysis of the Fluoroquinolone Resistant Mutant of the AcrB Transporter from Salmonella
Source: Microorganisms. 2020 Jun 23;8(6):943. doi: 10.3390/microorganisms8060943 (PMC7355581; doi:10.3390/microorganisms8060943)
Supplement: Supplementary file 1 [file microorganisms-08-00943-s001.pdf]

microorganisms

MDPI

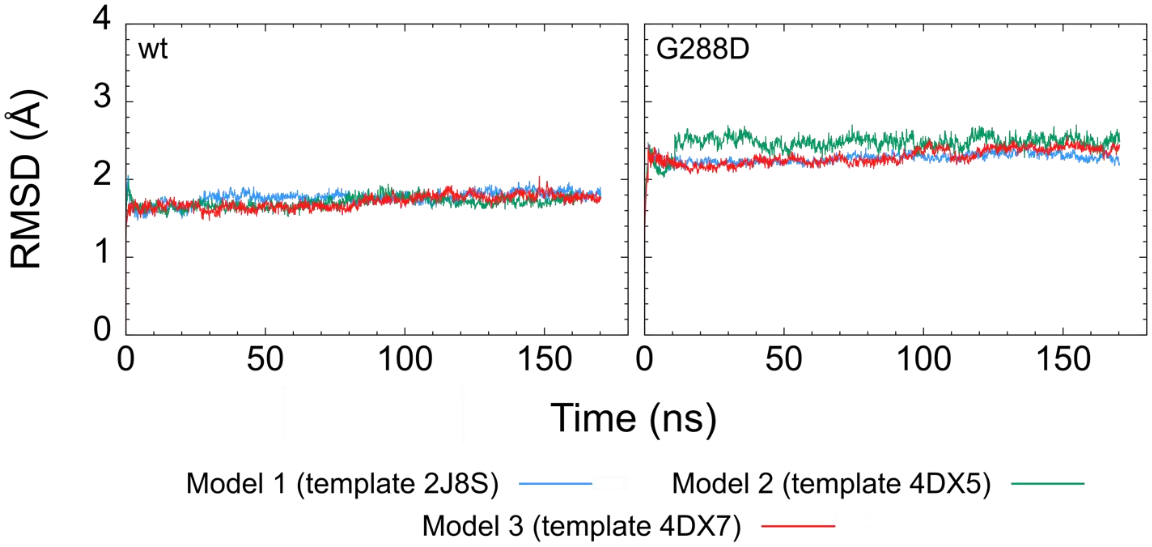

Figure S1. Protein RMSD calculated along the MD trajectories of *S*TmAcrB$_{WT}$ and *S*TmAcrB$_{G288D}$. Only the C$_{\alpha}$ atoms were considered.

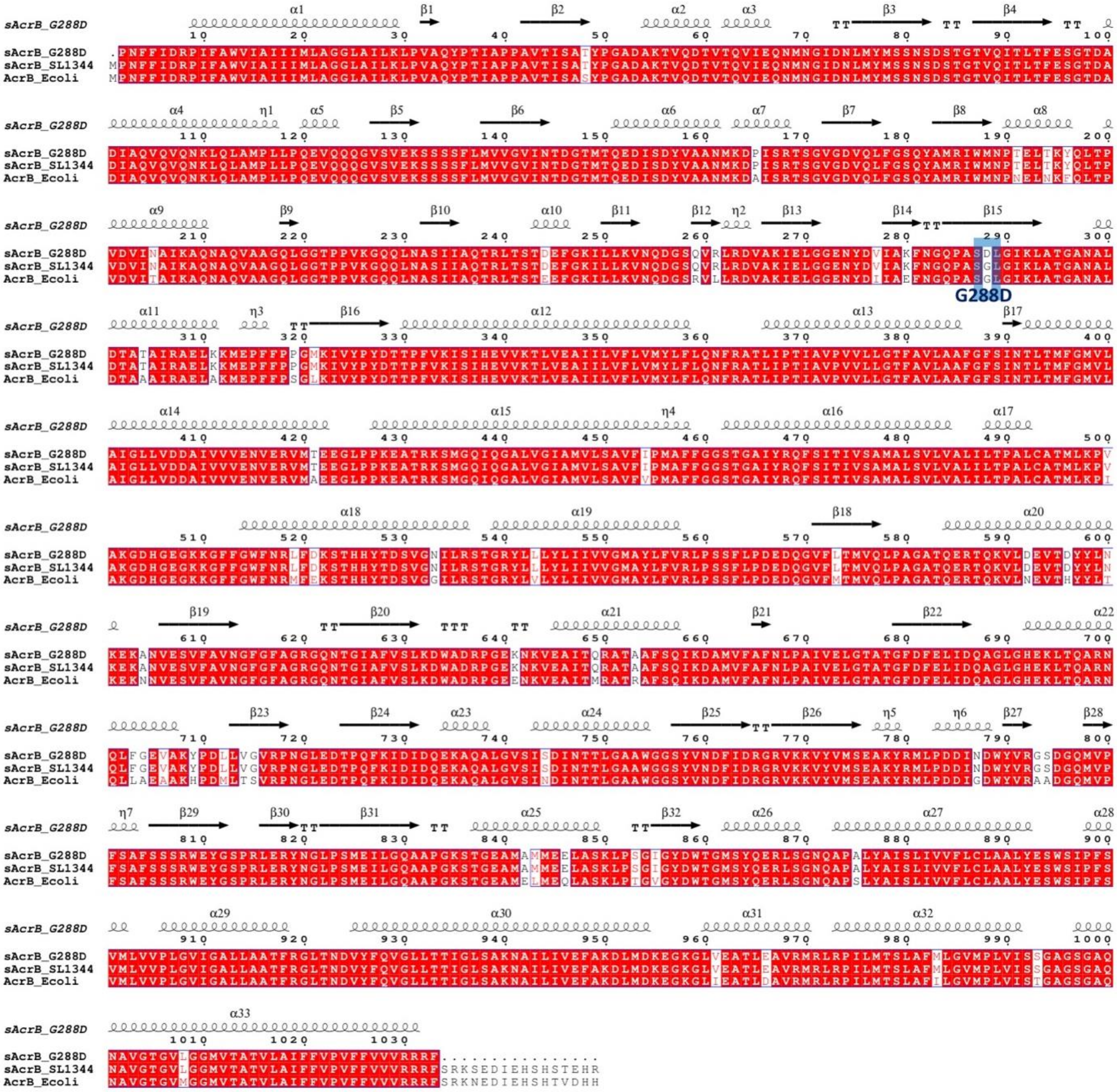

Figure S2. Multiple sequence alignment showing the secondary structure derived from *Salmonella* *S*TmAcrB$_{G288D}$ mutant cryo-EM structure reported here (top) aligned to the *S*TmAcrB$_{WT}$ from *Salmonella* SL1344 (Uniprot A0A0H3N916) and wild type AcrB from *E. coli* K12 (Uniprot P31224). The position of the G288D substitution is highlighted by a blue frame.

Table S1. Data collection, processing and model fitting statistics.

Data collection/ processing parameter	*S*TmAcrB$_{G288D}$ (C3)
Magnification	**130,000 x**
Voltage (kV)	**300**
Electron exposure (e-/Å2)	**61.7**
Defocus range (μm)	**-1.5 to -4.5**
Pixel size	**1.065**
Number of Micrographs	**3210**
Final particle number	**105,901**
FSC threshold	**0.143**
Map resolution (Å)	**4.6**
**Model Refinement**	
Poor Rotamers	**4 (0.17%)**
Favoured rotamers	**2302 (98.8%)**
Ramachandran outliers	**3 (0.1%)**
Molprobity Score	**2.44**

Table S2: Values of the Cross-Correlation Function obtained through Flex-EM [51] for the homology models of *S*TmAcrB$_{G288D}$, before and after the optimization inside the cryo-EM map. The RMSD of the optimized models with respect to the starting ones is also reported in the last column (only the C_{α} atoms were considered for this calculation).

Template [PDB ID]	CCF$_{init}$ [a.u.]	CCF$_{final}$ [a.u.]	RMSD$_{init/final}$ [Å]
2J8S	0.73	0.75	1.2
4DX5	0.73	0.75	1.1
4DX7	0.73	0.75	1.2
